# High acceptability of rapid HIV self-testing among a diverse sample of MSM from Buenos Aires, Argentina

**DOI:** 10.1371/journal.pone.0180361

**Published:** 2017-07-03

**Authors:** Maria A. Pando, Curtis Dolezal, Rubén O. Marone, Victoria Barreda, Alex Carballo-Diéguez, Maria M. Avila, Ivan C. Balán

**Affiliations:** 1 CONICET-Universidad de Buenos Aires, Instituto de Investigaciones Biomédicas en Retrovirus y Sida (INBIRS), Buenos Aires, Argentina; 2 HIV Center for Clinical and Behavioral Studies, New York State Psychiatric Institute and Columbia University, New York, New York, United States of America; 3 Nexo Asociación Civil, Buenos Aires, Argentina; Katholieke Universiteit Leuven Rega Institute for Medical Research, BELGIUM

## Abstract

**Introduction:**

The objective of this study was to explore the acceptability of rapid HIV self-testing (RHST) among men who have sex with men (MSM).

**Methods:**

During 2006–2009, a sample of 500 MSM was recruited through Respondent Driven Sampling for an HIV prevalence/incidence study. Attitude toward RHST was explored among HIV negative MSM. Data were weighted prior to analyses.

**Results:**

Participants reported they were likely to buy RHST (74%), test themselves more frequently than they currently do (77%), and that the procedure would simplify testing (70%). Furthermore, 71% reported they would probably use it alone, 66% would use it with a steady partner, and 56% with a friend/partner. While a majority acknowledged that RHST use would deprive them of receiving counseling (61%), 74% declared they would go for help if they tested positive; 57% would use an RHST in order to avoid condoms. Probability of use surpassed 70% among gay and non-gay identified MSM as well as those with and without a previous HIV test. Those likely to buy RHST were older (p = 0.025) and more likely to identify as gay (p = 0.036). A total of 17% said they would think about killing themselves and 9% would attempt suicide if they tested positive. These MSM were more likely to be younger (p<0.001), with lower mood level (p<0.001) and greater feelings of loneliness (p = 0.026).

**Conclusions:**

The high acceptability of RHST found among MSM should encourage the authorities to consider the possibility of offering it for self-testing, as it can improve early diagnosis and prevention of future transmissions. However, further research is needed to understand how to best disseminate RHST among MSM who wish to use it and to offer support and linkage to care for those who test HIV-positive.

## Introduction

Previous studies performed in Argentina revealed that men who have sex with men (MSM) are at high risk of Human Immunodeficiency Virus (HIV) infection [1]. The last cross-sectional study estimated HIV prevalence at 17.3% and HIV incidence at 4.5 per 100 persons-year; as well as high frequency of other sexually transmitted infections (STIs) like hepatitis B (22.9%), hepatitis C (7.5%), *Treponema pallidum* (20.5%) and human papilloma virus (HPV) (83.5%) [2]. Additionally, the last epidemiological bulletin of the Ministry of Health in Argentina reported that approximately 25% of the MSM that were diagnosed with HIV in the last period studied (2012–2015) were diagnosed with an AIDS (Acquired Immune Deficiency Syndrome) disease marker [3].

Late diagnosis of HIV infection has undesirable consequences, both for the individuals and for the wider population. Individuals with an advanced stage of immunosuppression are at high risk of diseases and death [4] with a worse response to highly active antiretroviral therapy (HAART) [5]. Thus, with the complexity of these cases, treatment costs increase [6]. Undiagnosed HIV-infected individuals are also more likely to contribute to a higher rate of transmission than those who are aware of their status [7]. These results reveal that it is imperative to develop innovative HIV testing strategies that can address the epidemic.

Following scientific evidence that rapid HIV testing facilitates HIV diagnosis through its ease of use in community settings [8–10], providing almost immediate test results [11, 12], and high acceptability among users [13–15], in 2012, the United States Food and Drug Administration approved the over-the-counter sale of an oral fluid HIV test that allows individuals to perform a rapid HIV self-test at home [16, 17]. The kit provides a result in 20 minutes and results are interpreted following written instructions without the need of professional help. Since then, this and other rapid HIV self-tests, have become available through pharmacies, online purchase, and, at times, through free distribution, in the U.S., Canada, and a number of European countries. Studies show high acceptability of self-testing among MSM [18–27] and transgender women (TGW), who expressed particular interest in using it to test with sexual partners [28]. Studies have also shown that individuals are able to use oral as well as finger-prick rapid HIV tests correctly [26, 27] and that access to free self-tests leads to greater frequency of testing among MSM [29]. While the scientific literature on the acceptability and use of self-testing is increasing in relation to at-risk populations in the US, Europe and Africa, similar research among high risk populations in other regions of the world, including Latin America is in its infancy [25]. In Latin America, two small studies on the acceptability of self-testing have been conducted, both reporting high acceptability. One also found that MSM could use a blood-based self-test correctly, while the other study raised concerns about linkage to care following HIV positive results [30, 31]. However, both studies used convenience samples of MSM with large proportions of the participants with post-secondary education, an HIV test within the past year, and in one study [30], a majority who were gay-identified, thus limiting the generalizability of the findings to the broader population of MSM.

Understanding acceptability and potential use of self-testing among high risk populations in Latin America is critical, as the region has been a late adopter of rapid HIV testing compared to other regions of the world.

Rapid HIV testing is relatively new in Argentina, where it first became available for use at clinics or specific testing centers in 2013 [32]. Previously, it was only available for occupational exposures, at time of labor if no previous tests had been performed, or used in massive HIV testing campaigns during specific occasions, such as International AIDS Day. Otherwise, HIV testing consisted of the traditional two-visit test spanning a one or two week period. As such, data on the acceptability of rapid HIV testing among MSM in Argentina is very limited. A qualitative study that explored the acceptability of rapid HIV testing among MSM in Buenos Aires prior to its availability in testing centers revealed that receiving results in 20 minutes (compared to the standard two week waiting period) was viewed as the critical advantage by the great majority of participants [33]. Discussion in focus groups also revealed that RHST was perceived as an advantage because of “privacy” and “convenience.” In regards to privacy, participants expressed the advantage of not having to go into a clinic or hospital where they might be labeled as gay or where they might be seen by friends or family members and asked about their being there, challenges that they perceived to be particularly important if one was not “out” in regards to their sexual orientation. Convenience was mentioned in regards not only to the quick results, but also in regards to not having to go to a clinic and experiencing extensive waits to be seen by a provider. Underlying these results were significant concerns about stigma and the ability of RHST to help MSM avoid uncomfortable situations where they might be judged or outed during the testing encounter. However, some participants, especially those who were already diagnosed HIV positive, expressed significant concerns about impulsive suicidal behavior if someone received a positive result at-home while alone [33]. As such, respondents favored the availability of rapid testing at community based organizations. Although rapid testing in organizations would reduce the waiting period for results it would not address concerns about privacy or convenience as fully as the availability of RHST.

This study aims to contribute to the literature on acceptability of self-testing for HIV among MSM in Latin America. It focuses on MSM living in the Buenos Aires, Argentina metropolitan area. This area constitutes not only the city of Buenos Aires, which has an established gay presence with numerous gay-identified venues and organizations, but also the surrounding areas, which are of significantly lower socioeconomic status and resources and where gay venues and organizations do not exist. Data from this manuscript comes from Proyecto LINKS, a study of HIV prevalence and risk behavior among 500 MSM recruited through Respondent Driven Sampling (RDS) [34, 35] in Buenos Aires, Argentina which found that 50% of participants had never been tested for HIV and 17% had only been tested once [36]. Similar to other studies in Latin America, Africa, and Asia that have used RDS [37–40], the sample is remarkable for the diversity of MSM recruited, especially in regards to high percentages of participants identifying as bisexual or heterosexual. The objective of this study was to assess the acceptability of RHST, its possible use with sexual partners, and potential for suicidality following HIV positive test results among MSM in the greater Buenos Aires, Argentina area.

## Methods

### Participant recruitment and data collection

Participant recruitment methodology and data collection procedures have been previously described [34]. Briefly, RDS combines ‘‘snowball sampling” with a mathematical model to compensate for non-randomness of participant selection (see http://www.respondentdrivensampling.org for details). A total of 16 MSM were selected as seeds for the RDS, completed all study procedures and then received three coupons each to give to members of their networks who could also recruit up to three other participants until the target number of 500 MSM was reached. Recruitment took place between November 2007 and July 2009. In order to be eligible to participate, the study candidate had to self-identify as a man, be 18 years or older, have had sex with another man or a TGW in the previous six months, have had sex with another man or TGW at least 10 times in his lifetime, reside in the Buenos Aires metropolitan area, agree to provide a blood sample for HIV and STI testing, and attend an interview with the coupon given to him by a prior participant. Participants who qualified underwent a consent process and responded to a Web-based survey presented in Spanish that included, among other things, demographic information, history of HIV testing, HIV knowledge, and recent sexual behavior. Once the questionnaire was completed, participants received pre-HIV counseling and provided a blood sample for HIV testing. About two weeks later, they returned to the testing site to pick up the HIV test result and to receive post-test counseling [2].

### Instruments

To assess the attitudes toward rapid HIV testing, we used a self-administered instrument that was developed for this study. The questionnaire was preceded by a photograph of an individual swabbing their gums with the oral HIV rapid test and the following statement: “In the USA, a rapid test is available to detect HIV antibodies in oral samples. To perform the test, the person being tested for HIV gently swabs the device completely around the outer gums (as you can see in the picture), and inserts it into a vial containing a developer solution. After 20 minutes, the test device will indicate if HIV antibodies are present by displaying two reddish-purple lines in a small window in the device. In the USA, this test is available in clinics. However, in the future it could be available in drugstores and individuals could buy the test and use it at home, like the pregnancy test. Hot lines would be available in order to answer questions about HIV. Assuming that this test (or a similar one) were available in Buenos Aires to purchase at an accessible price, please indicate the probability of the following statements”.

**Fig 1:**
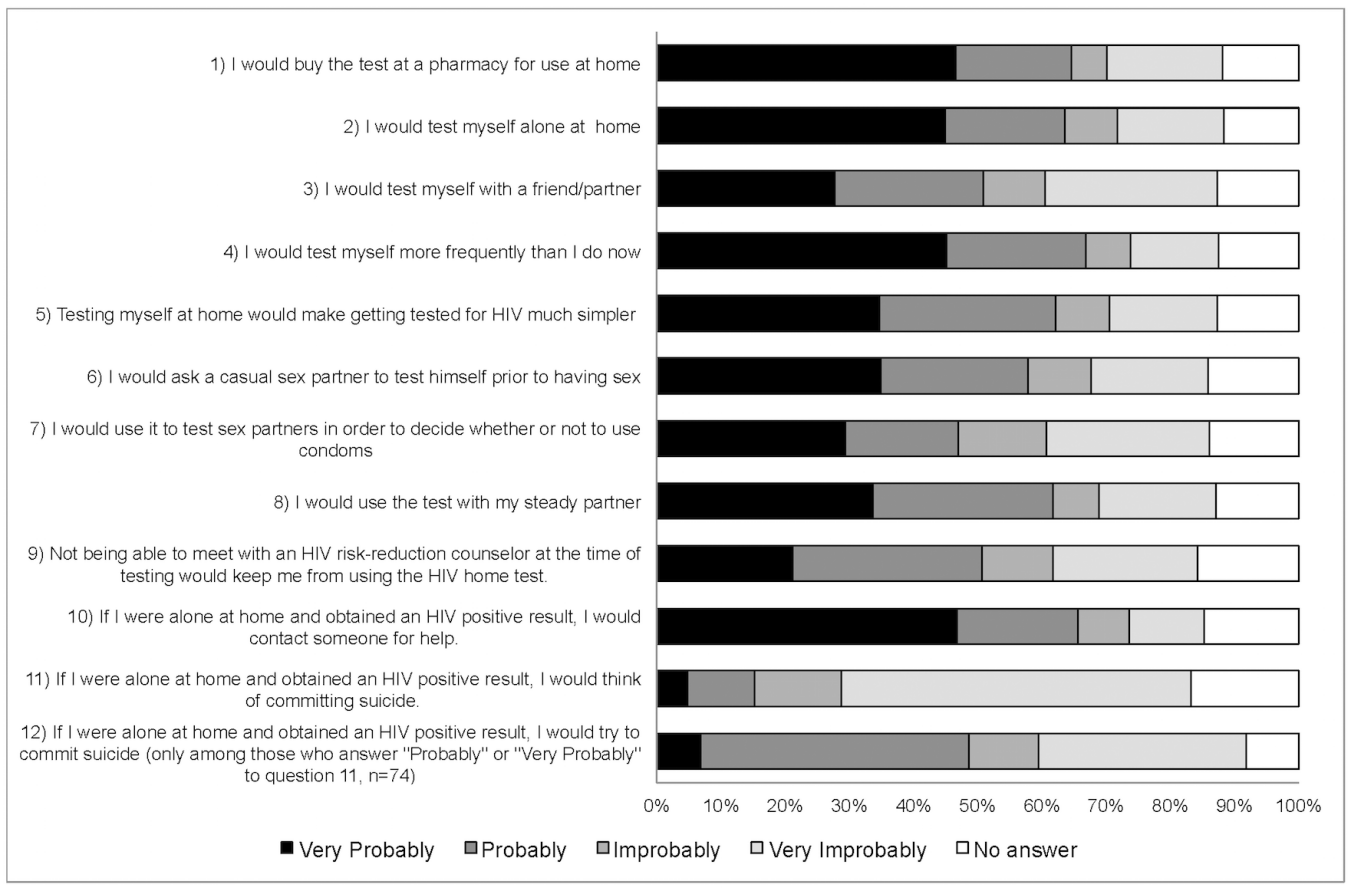
Likelihood of using RHST among MSM in Argentina. ^1^Frequency of “Very Probably”, “Probably”, “Improbably”, and “Very Improbably” vary due to missing data.

Then, the questionnaire addressed the probability of purchasing the RHST and doing the test under different circumstances (Fig 1). Participants answered items as “Very improbable,” “Improbable,” “Probable”, “Very probable” and “Prefer not to answer”. Participants were not able not able to skip questions. At the time of analysis, the first two categories were merged as “improbable,” and the second two as “probable.” Those participants who answered “Probable” or “Very Probable” to suicidal ideation, were asked two further questions: 1) the probability of them trying to commit suicide and 2) whether they had a prior suicide attempt.

### Ethics statement

International and national ethical guidelines for biomedical research involving human subjects were followed. This research study received approval from a local IRB (*Comité Independiente de Etica en Investigación* (CIEI-FM-UBA)) and the IRB of the New York State Psychiatric Institute, and was conducted in compliance with all federal regulations governing the protection of human subjects. All potential participants underwent an informed consent procedure prior to entering the study. Written consent was obtained from all participants prior to enrollment in the study.

### Data analysis

Unlike a conventional probability sampling design in which each unit has a known and constant probability of selection, in RDS each person sampled does not have the same probability of being included in the sample: rather, persons with larger personal networks have a greater likelihood of being sampled than those with smaller personal networks. RDS takes this into consideration by weighting data based on reported network size. Therefore, for all analyses, data were weighted prior to analyses using SPSS. The weighting estimator used is based on the RDS II estimator [41]. Weights were calculated as the inverse of the participant’s personal network size (PNS). This value was then multiplied by the sample size (N) divided by the sum of weights (∑w). The weighting formula was the following: (1/*PNS*) × (*N*/Σ*w*).

This provided results that reflect the original sample size of 500. Responses to each attitude question were dichotomized into Probable and Improbable groups. Two-group comparisons were then conducted using t-tests (for continuous variables), ANOVAS (for the categorical variable, "Region of city”), and chi-square tests (for dichotomous variables).

## Results

### Study population characteristics

A total of 500 MSM were recruited. Participants who self-reported being HIV positive and those who did not answer any of the RHST questions were excluded from analyses. After weighting the data, analyses were performed based on a final N of 404 men. Note that missing data (e.g., "Refuse to answer") was very common for the individual RHST questions, so Ns for any particular item do not sum to 404. Mean age was 30.3 years old (SD = 11.5). Only 24% of participants identified as gay; 37% identified as bisexual, 23% as heterosexual, and 16% were grouped as other. Thirty-seven percent reported having had sex with men, women, and TGW in the two months prior to the interview. Low formal education level was reported, including 66% of participants with incomplete high school. Thirty percent of participants reported being unemployed and 32% having a temporary job. Most participants were single (77%) and 82% had no health insurance. For a more detailed description of the population, please see Carballo-Dieguez et al. [34].

### Likelihood of using RHST

As seen in Fig 1, there was high acceptability of RHST. The great majority of participants (74%) reported that they would likely buy the test to use at home. Furthermore, 77% reported that they would test more often in the future if self-testing were available and 70% reported that it would be easier to do the test for HIV if they could do it at home. A majority of participants indicated they would probably use the test alone (71%), with a steady partner (66%), or with a friend/partner (56%). Lastly, 66% reported they would ask their casual partners to do the test before sex and 57% would use the test with their sex partners in order to avoid using of condoms. Probable use of RHST was high among gay (82%) and non-gay identified (71%) MSM as well as with those with (75%) and without (73%) a prior HIV test.

### Factors associated with RHST use

As shown in Table 1, likelihood of buying an RHST at a pharmacy for use at home was greater among older participants (p = 0.025) and those who were gay-identified (p = 0.036). Income, region of the city in which the participant resided (which affected availability of HIV testing sites), nor having a prior HIV test predicted the probability of purchasing a RHST.

**Table 1:** Factors associated with buying RHST at pharmacy for use at home among MSM in Argentina.

	Probable Use (N = 287)	Improbable Use (N = 103)	
	Mean (SD)	Mean (SD)	p-value^1^
Age	31.3 (11.5)	28.3 (11.6)	0.025
	N (%)^2^^-^^3^	N (%)^2^^-^^3^	p-value^1^
High School graduate	107 (38%)	26 (25%)	0.022
Low income	165 (70%)	55 (71%)	0.882
Region of city			
-Buenos Aires	78 (27%)	33 (32%)	0.743^3^
-West	148 (52%)	53 (52%)	
-South	51 (18%)	17 (16%)	
-North^4^	10 (3%)	0 (0%)	
Identify as gay	77 (27%)	17 (17%)	0.036
Prior HIV test	125 (44%)	42 (42%)	0.633

^1^ P-values from t-tests (age), ANOVAs (region of city), or Chi-square tests (dichotomous variables). Alpha for hypothesis testing is set at 0.05.

^2^ Ns do not always sum to 287 and 103 due to missing data.

^3^ Percents are of those with non-missing data.

^4^ "North" excluded from ANOVA due to small N.

### Suicidal tendencies

As seen in Fig 1, 63 participants (17%) reported that they would think of committing suicide if they self-tested at home and received a positive result (44 reported this was probable and 19 reported it was very probable). Of those 63, 32 (32/58 = 56%; 5 did not respond) indicated they would probably try to commit suicide (7 reported it would be very probable). Of these 32 participants, 11 (11/27 = 41%; 5 did not respond) reported a prior suicide attempt.

Factors associated with suicidal ideation (Table 2) include lower age (p<0.001), lower income (p = 0.014), low mood level (p<0.001), and feeling of loneliness (p = 0.030).

**Table 2:** Factors associated with probable suicidal ideation and probable suicidal action following HIV-positive results on a RHST among MSM in Argentina.

	Suicidal ideation			Suicidal action		
	Probable (n = 63)	Improbable (n = 303)		Probable (n = 32)	Improbable (n = 329)	
	Mean (SD)	Mean (SD)	p-value^1^	Mean (SD)	Mean (SD)	p-value^1^
Age	25.4 (7.7)	31.8 (11.9)	<0.001	25.0 (7.6)	31.5 (11.7)	<0.001
Mood Level	3.5 (0.8)	4.2 (0.8)	<0.001	3.5 (0.8)	4.2 (0.8)	<0.001
Mood Reactivity	3.8 (0.8)	3.7 (0.9)	0.137	4.0 (0.8)	3.7 (0.9)	0.063
Loneliness	2.4 (0.5)	2.2 (0.4)	0.030	2.4 (0.4)	2.2 (0.4)	0.026
	N (%)^2^	N (%)^2^	p-value^1^	N (%)^2^	N (%)^2^	p-value^1^
High School grad	22 (35%)	103 (34%)	0.915	13 (41%)	112 (34%)	0.470
Low income	44 (85%)	170 (68%)	0.014	23 (85%)	188 (69%)	0.073
Region of city						
-Buenos Aires	12 (19%)	93 (31%)	0.151^3^	10 (31%)	95 (29%)	0.277^3^
-West	39 (62%)	153 (50%)		13 (41%)	175 (53%)	
-South	11 (18%)	49 (16%)		8 (25%)	51 (16%)	
-North	1 (2%)	8 (3%)		1 (3%)	8 (2%)	
Identify as gay	14 (22%)	72 (24%)	0.793	10 (31%)	74 (23%)	0.267
Prior HIV test	29 (48%)	130 (43%)	0.546	13 (42%)	143 (44%)	0.814
Heavy Alcohol Use	19 (31%)	85 (29%)	0.773	12 (39%)	90 (28%)	0.211
Frequent Drug use	27 (44%)	130 (43%)	0.926	14 (44%)	142 (43%)	0.960

^1^ P-values from t-tests (continuous variables), ANOVAs (region of city), or Chi-square tests (dichotomous variables). Alpha for hypothesis testing is set at 0.05.

^2^ Percents are of those with non-missing data.

^3^ "North" excluded from ANOVA due to small N.

Factors associated with probable suicidal action following HIV positive results when alone were lower age (p<0.001), low mood level (p<0.001), and feeling lonely (p = 0.026). Neither educational level, identifying as gay, having had a prior HIV test, mood reactivity, heavy alcohol use, nor frequent drug use were significantly associated with probable suicidal ideation or probable suicidal action.

## Discussion

Findings of this first ever study of acceptability of HIV self-testing among MSM in Argentina not only demonstrate high acceptability of RHST among a highly diverse sample of MSM, but also suggest that the availability of self-testing could increase rates and frequency of HIV testing. The majority of participants also reported that they would test sexual partners, both steady partners as well as casual, allowing for further dissemination of RHST through high-risk sexual networks. Furthermore, high acceptability among MSM, regardless of sexual identity and prior HIV testing history, suggests that the availability of RHST may reach MSM who previous research has shown do not undergo HIV testing with sufficient frequency to allow for early detection of infection, entry into treatment, and protection of sexual partners—especially among non-gay-identified MSM [34]. This implies that the population with the most urgent need to test may be reached by the availability of RHST, thus filling an important gap by decreasing late diagnoses. This is critical, as the failure of early HIV diagnosis represents an important public health problem worldwide [42,43]. Reducing the number of undiagnosed individuals is the first step in expanding care and treatment, viral suppression, and ultimately in preventing subsequent infection to new partners.

Greater probability of use by older as well as gay-identified respondents may suggest greater comfort in dealing with results and greater availability of supports and knowledge of resources than may exist among younger or non-gay identified MSM. The absence of difference in acceptability based on region of Buenos Aires is encouraging, as the areas surrounding Buenos Aires have fewer resources and testing sites. Thus, free or low-cost distribution of RHST in these areas could greatly facilitate HIV testing among its residents since they would be able to access testing without the associated costs and time required to travel to the center of the city. Furthermore, RHST would also allow these MSM to avoid HIV testing in hospital settings where they may be concerned about stigmatization or being seen by acquaintances or family members. These challenges are similar to those faced by rural populations, for whom home testing and RHST have been proposed as approaches to facilitate HIV testing [44–46].

One of the most common concerns regarding self-testing focuses on the absence of a face-to-face counselor that can provide emotional support in the event of a positive result. In our study, approximately 17% of participants reported they would think of committing suicide if they obtained an HIV positive result using a RHST while alone, with half of those reporting they would probably attempt suicide in this circumstance. Our analyses revealed that these participants were more likely to be young, lonely, and experiencing dysphoric mood. A high proportion of these individuals also had a prior history of suicide attempts. This suggests that this group of individuals includes an important proportion who would have resorted to suicide in response to stressors, not only for the possibility of HIV-infection. These findings, however, raise the question as to whether MSM in lower or middle-income countries whose life situation may limit their access to HIV knowledge, support, and treatment, as well as emotional wellbeing, may be at risk for suicidality following HIV positive self-test results. Concerns about suicidality following home testing arose in the U.S. when HIV home tests first became available in the 1990s. However, there has been little evidence of suicidal attempts following HIV self-testing [19, 47, 48]. This suggests that respondents’ anticipated reactions to HIV-positive test results may not be reflective of their actual reactions. It is also possible that MSM who may feel emotionally unprepared to self-test at home alone would not opt to purchase an HIV self-test [49] and instead access HIV testing through other means (i.e., in hospitals, clinics, community organizations) where they may feel more comfortable. Nonetheless, studies of acceptability of RHST among populations in low- and middle-income countries may find it prudent to explore this issue among their population, including how HIV stigma and homophobia may affect suicidal ideation or action following positive test results. Such insights into how potential users may respond to HIV-positive test results may guide dissemination approaches. For example, rather than make RHST available through pharmacies, they may be available through HIV testing organizations, which can establish a provider relationship with the user in case support is needed during or after the self-testing process. As such, it is important to understand the psychosocial factors that may affect the choices made in regards to HIV testing practices and approaches, as these may vary across different subsets of MSM [50]. These insights can inform future studies on the acceptability of various dissemination approaches (ie., purchased in pharmacies, distributed through clinics or community organizations) and assess the impact of accessibility to self-tests on the frequency and rates of HIV testing among high risk populations.

Findings from this study should be considered in light of limitations. First, although RDS is considered an effective technique to sample most-at-risk populations, some publications have raised questions about its suitability for key aspects of public health surveillance [51] as well as the actual representativeness of the sample [52]. Second is that the data was collected in 2007–2009 and although the use of rapid HIV testing in testing centers has been rolled out over the past three years, the data may not reflect current views towards RHST. For example, since the introduction of rapid HIV testing at the community agency where this study was conducted the number of HIV test conducted per year have doubled. Thus, increasing awareness and experience with rapid testing may further increase the acceptability of RHST. Lastly, acceptability of product use does not always ensure use of the product in real life. Nonetheless, given the limited data available on acceptability of RHST in Latin America, a region that has trailed the world in the widespread availability of rapid HIV testing, these findings contribute to an emerging understanding of the acceptability of RHST among high risk groups in this region. Furthermore, consistent with the findings by Balán, et al [33], these results highlight high acceptability of novel HIV testing technologies that minimize the burden of HIV testing by increasing immediacy of results, convenience, and privacy. Considering the different methodologies available for self-testing, including type of samples (e.g. oral vs. blood sample test), and waiting time (from one minute to half an hour), and also the different approaches to accessing RHST (e.g. over-the-counter sales, distribution through HIV testing centers or community-based organizations, online purchasing) a critical next step is to explore user preferences in type of test and dissemination approaches to maximize the use of RHST among MSM in Argentina and, potentially, other Latin American countries.
